# End-stage renal disease in young black males in a black-white population: longitudinal analysis of the Bogalusa Heart Study

**DOI:** 10.1186/1471-2369-10-40

**Published:** 2009-12-02

**Authors:** Paul Muntner, Asghar Arshad, Stephen A Morse, Dharmendrakumar A Patel, Pronabesh D Manapatra, Efrain Reisin, Erwin A Aguilar, Wei Chen, Sathanur Srinivasan, Gerald S Berenson

**Affiliations:** 1 University of Alabama at Birmingham, Birmingham, AL, USA; 2 Louisiana State University Health Sciences Center, New Orleans, LA, USA; 3 Tulane University Health Sciences Center, New Orleans, LA, USA

## Abstract

**Background:**

Risk factors in childhood create a life-long burden important in the development of cardiovascular (CV) disease in adulthood. Many risk factors for CV disease (e.g., hypertension) also increase the risk of renal disease. However, the importance of childhood risk factors on the development of chronic kidney disease and end-stage renal disease (ESRD) is not well characterized.

**Methods:**

The current observations include data from Bogalusa Heart Study participants who were examined multiple times as children between 1973 and 1988.

**Results:**

Through 2006, fifteen study participants subsequently developed ESRD in adulthood; seven with no known overt cause. Although the Bogalusa Heart Study population is 63% white and 37% black and 51% male and 49% female, all seven ESRD cases with no known overt cause were black males (p < 0.001). Mean age-adjusted systolic and diastolic blood pressure in childhood was higher among the ESRD cases (114.5 mmHg and 70.1 mmHg, respectively) compared to black (103.0 mmHg and 62.3 mmHg, respectively) and white (mean = 103.3 mmHg and 62.3 mmHg, respectively) boys who didn't develop ESRD. The mean age-adjusted body mass index in childhood was 23.5 kg/m^2 ^among ESRD cases and 18.6 kg/m^2 ^and 18.9 kg/m^2 ^among black and white boys who didn't develop ESRD, respectively. Plasma glucose in childhood was not significantly associated with ESRD.

**Conclusion:**

These data suggest black males have an increased risk of ESRD in young adulthood. Elevated body mass index and blood pressure in childhood may increase the risk for developing ESRD as young adults.

## Background

There are multiple underlying causes of chronic kidney disease leading to end-stage renal disease (ESRD). However, there are few long term observations on risk factors in childhood for ESRD in adulthood. Such observations are important from the standpoint of prevention as the number of patients with ESRD treated with renal replacement therapy, dialysis or transplantation, has been increasing in the US and worldwide [1,2]. The number of incident and prevalent ESRD cases in the US is projected to rise from 93,000 and 382,000, respectively, in 2000 to 136,000 and 712,000, respectively, by 2015 [3]. Therefore, understanding the early natural history of ESRD is of considerable interest.

Epidemiologic studies have demonstrated that cardiovascular (CV) risk factors, such as hypertension and diabetes mellitus, are identifiable in childhood and are predictive atherosclerotic vascular disease in adulthood [4-8]. Autopsy studies in youth have also established a strong association between CV risk factors and early stages of coronary atherosclerosis [9-13]. These observations have also shown renal vasculature to be involved at a young age. Our autopsy studies [9-12] and the earlier work of Folkow et al [14] readily showed "adaptive structural changes of the vascular walls in hypertension" that can be occurring in youth and influence vascular resistance[15]. In addition, non-invasive studies of structure/function of the CV system are now able to show subtle changes begin in childhood that are strongly associated with clinical CV risk factors.

In adults, multiple CV risk factors are known to be associated with the development of renal disease [16-19]. However, few observations have been made on the association of such factors measured in childhood with the development of ESRD in young adulthood. The purpose of the current study is to note determinants obtained through a general risk factor screenings in childhood, adolescence and young adulthood with the occurrence of ESRD in young adulthood.

## Methods

### Study Population

The Bogalusa Heart Study, beginning in 1973, consists of multiple cross-sectional examinations of children residing in the bi-racial (65 percent white, 35 percent black) community of Bogalusa, Louisiana[8]. These individuals were followed by screening into young adulthood. The current study cohort includes those who participated in at least one examination as a child in 1973-1974 (n = 3865), 1975-1976 (n = 4222), 1981-1982(n = 2832), 1984-1985(n = 2149), and1987-1988 (n = 2582). The vast majority of participants were less than 18 years of age at baseline with only 28 participants ≥ 20 years of age. For participants with multiple examinations, data from various visits will be presented along with extended data obtained from clinic records at the community public hospital.

### General Examination

As described elsewhere, all Bogalusa Heart Study examinations followed the same protocols and procedures[8,20]. Interview data collected in the study visits included demographic questions and a medical history. Of relevance to the current analysis, each participant was asked whether they had been previously diagnosed with diabetes mellitus or kidney disease. Height and weight were measured twice to the nearest 0.1 cm and 0.1 kg, respectively, and averaged for analysis. Body mass index (BMI) was calculated using weight in kilograms divided by height in meters squared. Three replicate systolic and diastolic blood pressure readings were measured by each of two randomly assigned trained observers using mercury sphygmomanometers on the right arm of participants in a relaxed sitting position. Average values of the six measurements were used for analyses. The fourth Korotkoff phase was used for diastolic blood pressure for children.

### Identification of end-stage renal disease cases

Initially, a listing of Bogalusa Heart Study participants was provided to the medical director of the dialysis clinic in Bogalusa, Louisiana. Eleven cases were identified. Also, a listing of the names, sex, date of birth, and the home address at the time of their Bogalusa Heart Study visit was provided to research staff at the United States Renal Data System (USRDS). The USRDS is a registry of all patients initiating treatment for ESRD in the United States. Unfortunately, social security numbers, all changes of names and addresses over the interval of the study were not available to facilitate matching to the USRDS. However, three additional ESRD cases and one from the local public hospital were identified resulting in 15 ESRD cases who originally participated in the Bogalusa Heart Study in childhood.

Bogalusa Heart Study participants gave informed consent at each examination. For those under 18 years of age, consent of a parent/guardian was obtained. Study protocols were approved by the Institutional Review Boards of the Louisiana State University and Tulane University Health Sciences Center.

### Statistical Methods

Characteristics of the Bogalusa Heart Study participants were recorded for each study visit included in the current analyses. These parameters include age, race, sex, systolic and diastolic blood pressure, height, weight, BMI, plasma glucose, and a previous diagnosis of diabetes mellitus and kidney disease.

Although cut-points for defining high blood pressure and high BMI are available for children, since these variables vary at different age periods and growth of child, we used age-sex-height specific percentiles for blood pressure and age-sex percentiles for BMI from each Bogalusa Heart Study visit the subjects attended. Of the ESRD cases identified, seven (all black males) had no specific or obvious etiologic cause of renal disease during childhood. The age-adjusted mean systolic and diastolic blood pressure, BMI, and plasma glucose were calculated and compared for cases and white and black male Bogalusa Heart Study participants who did not develop ESRD, separately, using generalized estimating equations accounting for multiple study visits attended. Statistical analyses were conducted using SAS 9.1 (Cary, NC).

## Results

As shown in Table 1, at the Bogalusa Heart Study visits included in the current analysis, on average children were 11.4 years of age, 37% were black, and 49% were females. In childhood, mean systolic and diastolic blood pressure were 102.7 mmHg and 63.0 mmHg, respectively, the mean BMI was 18.7 kg/m^2^, and the mean plasma glucose was 84.1 mg/dL. A diagnosis of diabetes and kidney disease was reported for 1.7% and 1.3% of children, respectively, at their Bogalusa Heart Study visit.

**Table 1:** Characteristics of Bogalusa Heart Study childhood participants, overall and by Bogalusa Heart Study visit.

	Overall	Year of Bogalusa Heart Study visit				
		
	Mean (SD)	1972-1973 (n = 3865)	1975-1976 (n = 4222)	1980-1981 (n = 3705)	1982-1983 (n = 3430)	1985-1986 (n = 2666)
Mean age, years	11.4 (3.6)	10.6 (3.1)	11.7 (3.9)	11.3 (3.8)	11.0 (3.7)	13.0 (2.8)

% black	37.0	37.1	35.9	37.1	38.2	37.0

% female	48.8	47.1	48.2	48.9	49.8	50.5

Mean SBP, mmHg	102.7 (11.0)	100.6 (10.1)	101.7 (11.3)	103.5 (11.2)	103.2 (10.8)	105.9 (10.6)

Mean DBP, mmHg	63.0 (9.0)	62.7 (8.1)	62.1 (10.1)	63.7 (8.6)	63.3 (8.6)	63.7 (8.7)

Mean height, centimeters	145.0 (20.0)	141.2 (18.9)	145.6 (20.9)	143.7 (20.6)	142.8 (20.5)	154.0 (15.0)

Mean weight, kilograms	41.4 (17.5)	37.2 (15.2)	41.9 (17.6)	40.5 (17.5)	40.2 (17.6)	49.5 (17.0)

Mean body mass index, kg/m^2^	18.7 (4.0)	17.8 (3.5)	18.7 (3.9)	18.6 (3.9)	18.7 (4.0)	20.3 (4.5)

Plasma glucose, mg/dL	84.1 (10.3)	***	88.8 (11.9)	81.3 (9.5)	79.9 (7.6)	85.2 (9.3)

% with diabetes	1.7	4.2	1.1	0.2	0.2	0.2

% with kidney disease	1.3	1.2	0.2	0.9	0.3	0.3

Table 2 shows observations of the fifteen Bogalusa Heart Study participants who were identified as having developed ESRD. Four children reported having diabetes or kidney disease at their Bogalusa Heart Study visit and an additional four ESRD cases had a known causes of ESRD based on the review of their hospital chart. For the seven presumably "healthy" black males during childhood who developed ESRD the associated study population was comprised of 37% black, 49% male and 19% black and male, respectively. Given this race-sex distribution, the probability of all seven ESRD cases being black is p < 0.001, male is p = 0.007, and black males is p < 0.001.

**Table 2:** Characteristics of Bogalusa Heart Study participants who developed end-stage renal disease (ESRD) in adulthood

			STUDY	AGE AT	AGE AT	BMI	SBP/DBP	GLUCOSE	COMORBID CONDITIONS
			
#	SEX	RACE	VISIT YEAR	VISIT	HEMODIALYSIS	(percentile)*	(percentile)*	(percentile)*	
**Participants with unknown ESRD etiology**									

1	MALE	BLACK	1977	12		26 (95)	104/68 (42/93)	93 (62)	

			1979	14		30 (98)	115/75 (59/76)	76 (20)	SMOKING

			1981	16		36 (99)	127/85 (91/94)	94 (96)	

			1989	24	34	36 (95)	136/88 (99/97)	81 (52)	HYPERTENSION

2	MALE	BLACK	1973	7		14 (8)	90/37 (30/1)		

			1975	9		14 (7)	90/40 (27/5)		3 + PROTEINURIA

			1977	10		15(11)	90/44 (17/3)	99 (92)	

			1979	13	38	15 (3)	99/64 (46/68)	83 (50)	

3	MALE	BLACK	1973	13		18 (44)	133/92 (100/100)		

			1977	17		22 (63)	135/84 (100/100)	102 (92)	SMOKING

			1989	29		25 (59)	137/95 (99/99)		

			1995	35		25 (35)	162/109 (100/100)	81 (61)	

			2002	42	43	28 (55)	184/134 (100/100)	76 (23)	

4	MALE	BLACK	1974	11		18 (62)	135/77 (100/100)		

			1979	16		21 (61)	139/92 (100/100)	72 (14)	

			1986	23		23 (48)	118/77 (81/84)	75 (15)	

			1988	25		23 (41)	120/71 (81/51)	74 (20)	

			1996	33	37	23 (16)	120/69 (64/33)		

5	MALE	BLACK	1974	11		25 (95)	118/66 (95/68)		

			1977	14		28 (94)	112/59 (66/11)	101 (80)	

			1979	16	Not available	32 (97)	132/82 (98/92)	84 (57)	SMOKING, OBESITY

6	MALE	BLACK	1973	13		15 (52)	132/78 (83/55)		

			1977	17		18 (38)	114/74 (42/74)	82 (14)	SMOKING

			1985	25		31 (93)	118/70 (83/52)	78 (29)	

			1989	29		35 (93)	138/79 (99/83)	90 (85)	

			1996	36		46 (99)	132/94 (95/98)	75 (33)	

			2001	41	44	25 (28)	113/70 (65/62)	85 (61)	

7	MALE	BLACK	1977	8		18 (85)	107/68 (93/91)	91 (77)	

			1979	10		21 (85)	104/66 (81/97)	97 (95)	

			1982	16		22 (80)	117/75 (95/80)	80 (40)	

			1984	18		25 (84)	129/77 (96/83)	83 (35)	

			1988	22	Not available	25 (55)	118/80 (77/87)	76 (27)	

**Participants with renal disease or diabetes during study visit in childhood**									

8	FEMALE	BLACK	1976	11		17 (46)	100/62 (20/21)	345 (100)	DIABETES

			1979	14		22 (66)	113/88 (70/100)	275 (100)	

			1989	24	39	22 (30)	98/68 (9/37)	413 (100)	SMOKING, OBESITY

9	FEMALE	BLACK	1974	6		20 (97)	115/70 (100/98)		

			1977	9		25 (97)	109/64 (91/75)		

			1979	11		28 (98)	110/71 (80/87)	81 (64)	SEVERE KIDNEY DISEASE, SMOKING

			1982	14		35 (98)	122/63 (95/43)	82 (50)	

			1989	21		37 (96)	113/65 (60/20)	257 (100)	

			1995	27	34	35 (87)	108/74 (45/77)	318 (100)	

10	MALE	WHITE	1973	7		20 (95)	102/64 (86/80)		

			1989	23	33	24 (49)	104/74 (24/65)	359 (100)	DIABETES

11	MALE	BLACK	1973	9		15 (19)	95.3/65 (44/80)		HEART DISEASE, KIDNEY DISEASE

			1977	13		20 (64)	107/77 (81/98)	86 (23)	SEVERE KIDNEY DISEASE

			1979	15		26 (90)	117/84 (96/100)	85 (29)	

			1984	20		21 (6)	134/80 (100/98)	84 (31)	

			1989	25		23 (37)	145/80 (100/85)	93 (91)	HBP

			2002	38	46	22 (11)		83 (53)	GLOMERULONEPHRITIS, RHEUMATIC FEVER

**Participants with other causes of renal disease**									

12	FEMALE	BLACK	1981	5		16 (19)	103/63 (44/26)	75 (51)	

			1983	7		16 (10)	95/52 (26/56)	77 (83)	

			1987	11		17 (9)	107/57 (27/58)	82 (57)	

			1992	16		26 (88)	127/74 (99/92)	74 (12)	LUPUS

					20				

13	MALE	WHITE	1974	16		27 (90)	126/77 (90/92)		SMOKING

					40				CONGENITAL SINGLE KIDNEY

14	MALE	WHITE	1973	15		31 (97)	127/79 (93/93)		

			1977	18		29 (93)	111/64 (37/25)	95 (71)	

			1989	30		30 (83)	150/99 (100/100)	86 (73)	TREATMENT FOR HYPERTENSION

			2002	43		34 (82)	123/82 (72/82)	95 (85)	

					39				GLOMERULONEPHRITIS, RENAL MALIGNANCY

15	FEMALE	BLACK	1974	13		22 (74)	110/66 (69/48)		

			1977	16		25 (81)	119/73 (80/55)	88 (11)	

			1985	24		21 (32)	136/95 (100/100)	75 (16)	HIV

			1988	27		20 (11)	138/117 (99/100)	88 (80)	TREATMENT FOR HYPERTENSION

* Percentiles derived from children of similar age during the same study visit

Of the seven ESRD cases with unknown etiology, as children, four had a BMI at or above their age-sex specific 80^th ^percentile during at least one of their Bogalusa Heart Study visits. Additionally, six of the seven ESRD cases had a systolic blood pressure or diastolic blood pressure above their age-sex-height specific 80^th ^percentiles at one or more of their Bogalusa Heart Study visits. Finally, six of the seven ESRD cases had a plasma glucose measurement at or above their age-sex-height specific 80^th ^percentile.

The seven ESRD cases with an unknown etiology during childhood attended 20 Bogalusa Heart Study visits throughout their childhood. The mean age of the cases was 13.3 and 11.4 and 11.9 years for white and black non-cases, respectively (Table 3). The age-adjusted mean systolic and diastolic blood pressure and BMI were each significantly higher among the cases when compared both with white and black non-cases, separately (each p < 0.001). In contrast, no statistically significant differences were present in the age-adjusted mean plasma glucose between cases and non-cases.

**Table 3:** Age and age-adjusted blood pressure, body mass index, and plasma glucose of ESRD cases with an un-identified cause and white and black non-cases.

	Cases (n = 7)	White non-cases (n = 3012)	Black non-cases (n = 1528)
**Number of study visits attended**	**20**	**6410**	**3749**

	**Mean (SE)**		

Age, years	13.3 (0.7)	11.4 (0.1)***	11.9 (0.1)***

SBP, mmHg^†^	114.5 (2.0)	103.3 (0.1)***	103.0 (0.1)***

DBP, mmHg^†^	70.1 (1.8)	62.3 (0.1)***	62.3 (0.1)***

BMI, kg/m^2†^	23.5 (0.8)	18.9 (0.4)***	18.6 (0.1)***

Plasma glucose, mg/dL^†^	87.0 (2.7)	85.8 (0.1)	84.0 (0.2)

Abbreviations: SBP - systolic blood pressure; DBP - diastolic blood pressure; BMI - body mass index.

All cases and controls are males. All cases are black males.

^† ^Adjusted to the age distribution of all Bogalusa Heart Study participants

* p < 0.05, ** p < 0.01, *** p < 0.001 comparing cases to controls

## Discussion

Cardiovascular disease risk factors have been associated with the development of renal disease in adults and the association of hypertension and obesity with CV target organ damage is now well recognized. Previous data from the Bogalusa Heart Study have demonstrated the importance of elevated blood pressure and BMI in childhood as predictors for the development of CV disease in adulthood[8,21-25]. The current study synthesizes these concepts by studying children who participated in the Bogalusa Heart Study and subsequently developed ESRD in adulthood. The occurrence of a selective preponderance of black males among ESRD cases with unknown etiology is highly unusual. Also, in the current study, a majority of the fifteen Bogalusa Heart Study participants identified as having developed ESRD in adulthood had elevated blood pressure and a high BMI in childhood but more of the causal or associated factors for driving black males particularly into ESRD needs to be elucidated. It is of interest that our autopsy studies showed medial thickening in small renal arteries (50-400 mm)[10,12] and renal vessel intima media changes that related to blood pressure levels by Tracy et al[26] all beginning at a young age, Folkow et al[14] years earlier pointed out that "adoptive structural changes of the vascular walls in hypertension are likely related to control of peripheral resistance". Since black children already have higher blood pressure levels, such renal artery changes might be expected to occur earlier in blacks[8,27].

Almost all patients with ESRD have hypertension. Whether hypertension precedes, or is a consequence of underlying renal disease still remains debatable. However, several longitudinal cohort studies have identified high blood pressure as a risk factor for the development and progression of renal dysfunction [28-31]. For example, using prospective follow-up from the Systolic Hypertension in the Elderly Program, Young and colleagues reported higher systolic blood pressure among older adults to be associated with a decline in kidney function (a rise in serum creatinine ≥0.4 mg/dl)[30]. More relevant to the current study, blood pressure has long been reported to be a risk factor for incident ESRD. Hsu and colleagues reported a strong graded relationship between higher blood pressure and ESRD risk among 316,675 men and women enrolled in the Kaiser Pemanente health plan of Northern California during 35 years of follow up[31]. Specifically, compared with adult patients, who had a systolic/diastolic blood pressure less than 120/80 mm Hg, the multivariate adjusted relative risks (95% confidence interval) for developing ESRD were 1.62 (1.27, 2.07), 1.98 (1.55, 2.52), 2.59 (2.07, 3.25), 3.86 (3.00, 4.96), 3.88 (2.82, 5.34), and 4.25 (2.63, 6.86) for their counterparts with a systolic/diastolic blood pressure of 120-129/80-84 mm Hg, 130-139/85-89 mm Hg, 140-159/90-99 mm Hg, 160-179/100-109 mm Hg, 180-209/110-119 mm Hg, and ≥210/120 mmHg. Even in the prehypertensive range[32,33], there is a projected increased risk of ESRD. The current study extends the previous findings in adults and shows childhood blood pressure at higher percentile levels, especially among black males, increases the risk of ESRD in young adulthood.

Diabetes mellitus in adults is also a major contributor to ESRD[34]. Also, with increasing obesity present in children, its role also has to be considered. BMI is an established risk factor for insulin resistance, the metabolic syndrome, diabetes mellitus and hypertension. Obesity in childhood is also associated with elevated blood pressure and adverse changes of CV risk factors, including changes in carbohydrate-insulin metabolism, especially in young white children. Thus, obesity is a major contributor to increase the risk of adult CV disease and ESRD [35-37]. The multivariable-adjusted relative risk for ESRD illustrated in the aforementioned study of Kaiser Permanente enrollees, compared with persons who had normal weight (BMI, 18.5 to 24.9 kg/m^2^), was 1.87 (95% CI, 1.64, 2.14),3.57 (3.05, 4.18), 6.12 (CI, 4.97, 7.54), and 7.07 (CI, 5.37, 9.31) for those with a BMI of 25.0 to 29.9 kg/m^2^, 30.0 to 34.9 kg/m^2^, 35.0 to 39.9 kg/m^2^, and ≥40 kg/m^2^. In the current study, BMI was significantly higher in children who later developed, compared to their counterparts who did not develop, ESRD. With regards to the CV problems, we have previously shown that BMI in childhood is the most consistent predictor of left ventricular mass and left ventricular dilatation in young adulthood[23,24]. The role of obesity may be through its metabolic pathway for hormonal, and inflammatory adipokine factors that influence renal structure and functional integrity, as well as hemodynamic factors noted for heart disease.

The occurrence of all seven ESRD cases of primarily unknown etiology being black males in a bi-racial cohort (63% white and 37% black) with an approximately equal gender distribution represents an unusual probability. However, this finding is consistent from US population data where the incidence of ESRD is more than 4-fold higher among young black, compared to white, US adults. In 2006, the last for which data are available, the incidence rate of ESRD among young US adults (20 to 39 years of age) was 341.7 and 265.3 for black males and females, respectively, and 79.8 and 57.7 for white males and females, respectively. The current observation explored the potential predictors of ESRD beginning in childhood and found higher BMI and higher levels of blood pressure in cases who developed ESRD compared with the white and black children and adolescents who did not develop ESRD. These risk factors were present even at the childhood age but we also note black males at the childhood age can have higher blood pressure levels even without obesity. Such observations show the complexity of understanding the early origin of ESRD and hypertensive disease. Other exposures like high dietary sodium and low potassium may be contributing factors to the development and acceleration of early hypertension[38,39]. Unfortunately, underlying, undetected chronic kidney disease leading to ESRD cannot be excluded. Histopathology of ESRD with hypertension and without diabetes has shown various forms of glomerulosclerosis[40]. Unfortunately the nature of renal glomerular pathology in young asymptomatic individuals with high percentiles of blood pressure levels, still within normal ranges has not been studied.

The group of children in this study who eventually developed ESRD is somewhat unusual when compared to the general adult ESRD population on dialysis. Beginning with a childhood population and trying to match those developing ESRD produced essentially 50% with a clinical etiology related to early and progressive renal disease from secondary causes. This group included a congenital abnormality of a single kidney, Type-I diabetes, lupus, and HIV. This diversity in the causes of ESRD is likely related to a selection beginning in childhood. Also, while BMI and blood pressure levels observed are still lower than those considered abnormal by adult standards, body mass, weight, and blood pressure in childhood track or persist into adulthood, and can be related to a CV-renal burden. Thus, identifying risk factors and emphasizing preventive measures beginning in childhood are highly germane.

The relevance of obesity to hypertension and ESRD needs specific emphasis. Among US children and adolescents, overweight has reached epidemic proportions[41]. According to data from the National Health and Nutrition Examination Surveys, overweight in children and adolescents increased from 5% in 1966-1970 to 15% in 1999-2002. Also, in the Bogalusa Heart Study, 32% of children 5 to 14 years of age and 37% of children 15-17 years of age examined in 1992 to 1994 would have been considered overweight based on a BMI above the 85th percentile from 1973-1974[42]. Although a number of abnormal risk factors have been linked to a high and increasing BMI in childhood[4,7] the current study indicates ESRD is yet another adverse health factor associated with childhood body fatness. Clearly, the prevention of overweight in children and adolescents remains an important public health challenge in the United States. However, decreasing BMI in children and young adults is a challenge. Several studies have been conducted in an attempt to reduce the burden of overweight and obesity. However, for the most part, these interventions have not proven successful. In Bogalusa, Louisiana, the "Health Ahead/Heart Smart" program has been developed and implemented. This program builds on more than two decade's research in creating an effective cardiovascular prevention and general health education for children. The goal of this program is to improve children's health and behavior using an approach that will last their entire life. Future studies are needed to evaluate the benefits of programs such as the "Health Ahead/Heart Smart" program on outcomes.

There are major limitations of the current study. One lies in the imperfect linkage between Bogalusa Heart Study data with the USRDS which could help detect additional individuals with ESRD. Specifically, social security numbers were not collected for children and adolescents at the time of their Bogalusa Heart Study visits in the 1970s and 1980s. Therefore, linkage to the USRDS relied on the Bogalusa Heart Study participants' names without changes, dates of birth and early street addresses. Clearly, the series of 15 cases being reported most likely represents only some of the ESRD cases that have occurred among Bogalusa Heart Study participants. Because ESRD among young adults is rare, the threat of bias to the current results is minimal. Perhaps an even greater limitation is the lack of a detailed follow up from childhood to young adulthood when ESRD occurred. A gap occurs in most cases between childhood risk factor surveys and hospital data.

## Conclusion

The current observations provide data showing an incidence of ESRD among black males higher than expected based on the race-sex composition of the Bogalusa Heart Study. Also, high blood pressure and BMI in childhood were associated with the development of ESRD in adulthood. In children, blood pressure and BMI levels have increased dramatically over the past 30 years. The association of these risk factors with ESRD along with subclinical evidence of changes to the CV renal system may account, in part, for the increasing incidence of ESRD and point to a critical area for prevention.

## Competing interests

The authors declare that they have no competing interests.

## Authors' contributions

PM, AA, SAM, ER, EAA, WC, SS, and GSB conceived and designed the study. DAP, PDM, WC, SS, GSB compiled and managed the patient database and were involved in data assembly. PM, DAP, and PDM performed the statistical analysis of the data. PM, AA, SAM, ER, EAA, and GSB drafted the article. ER, WC, SS were responsible for critical revision of the article for important intellectual content. All authors read and approved the final manuscript. PM and GSB are the guarantors of the integrity of the study.

## Pre-publication history

The pre-publication history for this paper can be accessed here:

http://www.biomedcentral.com/1471-2369/10/40/prepub

## References

[B1] Meguid El NA Bello AK Chronic kidney disease: the global challenge Lancet 2005 365 331 340 15664230 10.1016/S0140-6736(05)17789-7

[B2] United States Renal Data System (USRDS) USRDS 2005 Annual Data Report: Atlas of End-Stage Renal Disease in the United States 2006 Bethesda, MD, National Instistutes of Health, National Institute of Diabetes and Digestive and Kidney Diseases

[B3] Gilbertson DT Liu J Xue JL Louis TA Solid CA Ebben JP Collins AJ Projecting the number of patients with end-stage renal disease in the United States to the year 2015 J Am Soc Nephrol 2005 16 3736 3741 10.1681/ASN.2005010112 16267160

[B4] Freedman DS Khan LK Serdula MK Dietz WH Srinivasan SR Berenson GS Inter-relationships among childhood BMI, childhood height, and adult obesity: the Bogalusa Heart Study Int J Obes Relat Metab Disord 2004 28 10 16 10.1038/sj.ijo.0802544 14652621

[B5] Hoq S Chen W Srinivasan SR Berenson GS Childhood blood pressure predicts adult microalbuminuria in African Americans, but not in whites: the Bogalusa Heart Study Am J Hypertens 2002 15 1036 1041 10.1016/S0895-7061(02)03066-2 12460698

[B6] Tzou WS Douglas PS Srinivasan SR Bond MG Tang R Chen W Berenson GS Stein JH Increased subclinical atherosclerosis in young adults with metabolic syndrome:the Bogalusa Heart Study J Am Coll Cardiol 2005 46 457 463 10.1016/j.jacc.2005.04.046 16053958

[B7] Chen W Bao W Begum S Elkasabany A Srinivasan SR Berenson GS Age-related patterns of the clustering of cardiovascular risk variables of syndrome X from childhood to young adulthood in a population made up of black and white subjects: the Bogalusa Heart Study Diabetes 2000 49 1042 1048 10.2337/diabetes.49.6.1042 10866058

[B8] Berenson GS McMahan C Voors AW Webber LS Srinivasan SR Frank GC Cardiovascular risk factors in children: The early natural history of atherosclerosis and essential hypertension 1980 New York, NY:Oxford University Press

[B9] Newman WP Wattigney W Berenson GS Autopsy studies in United States children and adolescents. Relationship of risk factors to atherosclerotic lesions Ann N Y Acad Sci 1991 623 16 25 10.1111/j.1749-6632.1991.tb43715.x 2042824

[B10] Newman WP Freedman DS Voors AW Gard PD Srinivasan SR Cresanta JL Williamson GD Webber LS Berenson GS Relation of serum lipoprotein levels and systolic blood pressure to early atherosclerosis. The Bogalusa Heart Study N Engl J Med 1986 314 138 144 3455748 10.1056/NEJM198601163140302

[B11] Tracy RE Newman WP Wattigney WA Berenson GS Risk factors and atherosclerosis in youth autopsy findings of the Bogalusa Heart Study Am J Med Sci 1995 310 Suppl 1 S37 S41 7503122 10.1097/00000441-199512000-00007

[B12] Tracy RE Mercante DE Moncada A Berenson G Quantitation of hypertensive nephrosclerosis on an objective rational scale of measure in adults and children Am J Clin Pathol 1986 85 312 318 3751980 10.1093/ajcp/85.3.312

[B13] McMahan CA McGill HC Gidding SS Malcom GT Newman WP Tracy RE Strong JP PDAY risk score predicts advanced coronary artery atherosclerosis in middle-aged persons as well as youth Atherosclerosis 2007 190 370 377 10.1016/j.atherosclerosis.2006.02.008 16530772

[B14] Folkow B Grimby G Thulesius O Adaptive structural changes of the vascular walls in hypertension and their relation to the control of the peripheral resistance Acta Physiol Scand 1958 44 255 272 10.1111/j.1748-1716.1958.tb01626.x 13617022

[B15] Mulvany MJ The fourth Sir George Pickering memorial lecture. The structure of the resistance vasculature in essential hypertension J Hypertens 1987 5 129 136 10.1097/00004872-198704000-00001 3302037

[B16] Brancati FL Whelton PK Randall B Neaton J Stamler J Klag MJ Risk of end-stage renal disease in diabetes mellitus: A prospective cohort study of men screened for MRFIT JAMA 1997 278 2069 2074 10.1001/jama.278.23.2069 9403420

[B17] Perneger TV Brancati FL Whelton PK Klag MJ End-stage renal disease attributable to diabetes mellitus Annals of Internal Medicince 1994 121 912 918 10.7326/0003-4819-121-12-199412150-00002 7978716

[B18] Muntner P Coresh J Smith JC Eckfeldt JH Klag MJ Plasma lipids and a decline in renal function: The Atherosclerosis Risk in Communities Study Kidney International 2000 58 293 301 10.1046/j.1523-1755.2000.00165.x 10886574

[B19] Sarnak MJ Coronado BE Greene T Wang SR Kusek JW Beck GJ Levey AS Cardiovascular disease risk factors in chronic renal insufficiency Clin Nephrol 2002 57 327 335 12036190 10.5414/cnp57327

[B20] Berenson GS Srinivasan SR Emergence of obesity and cardiovascular risk for coronary artery disease: the Bogalusa Heart Study Prev Cardiol 2001 4 116 121 10.1111/j.1520-037X.2001.00537.x 11828187

[B21] Chen W Srinivasan SR Elkasabany A Berenson GS Cardiovascular risk factors clustering features of insulin resistance syndrome (Syndrome X) in a biracial (Black-White) population of children, adolescents, and young adults: the Bogalusa Heart Study Am J Epidemiol 1999 150 667 674 10512420 10.1093/oxfordjournals.aje.a010069

[B22] Srinivasan SR Bao W Wattigney WA Berenson GS Adolescent overweight is associated with adult overweight and related multiple cardiovascular risk factors: the Bogalusa Heart Study Metabolism 1996 45 235 240 10.1016/S0026-0495(96)90060-8 8596496

[B23] Haji SA Ulusoy RE Patel DA Srinivasan SR Chen W Delafontaine P Berenson GS Predictors of left ventricular dilatation in young adults (from the Bogalusa Heart Study) Am J Cardiol 2006 98 1234 1237 10.1016/j.amjcard.2006.05.054 17056336 PMC3228635

[B24] Li X Li S Ulusoy E Chen W Srinivasan SR Berenson GS Childhood adiposity as a predictor of cardiac mass in adulthood: the Bogalusa Heart Study Circulation 2004 110 3488 3492 10.1161/01.CIR.0000149713.48317.27 15557363

[B25] Burke GL Arcilla RA Culpepper WS Webber LS Chiang YK Berenson GS Blood pressure and echocardiographic measures in children: the Bogalusa Heart Study Circulation 1987 75 106 114 2947739 10.1161/01.cir.75.1.106

[B26] Tracy RE Velez-Duran M Heigle T Oalmann MC Two variants of nephrosclerosis separately related to age and blood pressure Am J Pathol 1988 131 270 282 3358455 PMC1880608

[B27] Voors AW Foster TA Frerichs RR Webber LS Berenson GS Studies of blood pressures in children, ages 5-14 years, in a total biracial community: the Bogalusa Heart Study Circulation 1976 54 319 327 939029 10.1161/01.cir.54.2.319

[B28] Perry H Miller J Fornoff J Baty J Sambhi M Rutan G Moskowitz D Carmody S Early Predictors of 15-Year End-Stage Renal Disease in Hypertensive Patients Hypertension 1995 25 587 594 7721402 10.1161/01.hyp.25.4.587

[B29] Vupputuri S Batuman V Muntner P Bazzano LA Lefante JJ Whelton PK He J Effect of blood pressure on early decline in kidney function among hypertensive men Hypertension 2003 42 1144 1149 10.1161/01.HYP.0000101695.56635.31 14597644

[B30] Young JH Klag MJ Muntner P Whyte JL Pahor M Coresh J Blood pressure and decline in kidney function: findings from the Systolic Hypertension in the Elderly Program (SHEP) J Am Soc Nephrol 2002 13 2776 2782 10.1097/01.ASN.0000031805.09178.37 12397049

[B31] Hsu CY McCulloch CE Darbinian J Go AS Iribarren C Elevated blood pressure and risk of end-stage renal disease in subjects without baseline kidney disease Arch Intern Med 2005 165 923 928 10.1001/archinte.165.8.923 15851645

[B32] Reynolds K Gu D Muntner P Kusek JW Chen J Wu X Duan X Chen CS Klag MJ Whelton PK He J A population-based, prospective study of blood pressure and risk for end-stage renal disease in China J Am Soc Nephrol 2007 18 1928 1935 10.1681/ASN.2006111199 17475822

[B33] Klag MJ Whelton P Randall B Neaton J Brancati F Ford C Shulman N Stamler J Blood pressure and End-stage renal disease in men NEJM 1996 334 13 18 10.1056/NEJM199601043340103 7494564

[B34] Mokdad AH Ford ES Bowman BA Dietz WH Vinicor F Bales VS Marks JS Prevalence of obesity, diabetes, and obesity-related health risk factors, 2001 JAMA 2003 289 76 79 10.1001/jama.289.1.76 12503980

[B35] Kramer H Luke A Bidani A Cao G Cooper R McGee D Obesity and prevalent and incident CKD: the Hypertension Detection and Follow-Up Program Am J Kidney Dis 2005 46 587 594 10.1053/j.ajkd.2005.06.007 16183412

[B36] Hsu CY McCulloch CE Iribarren C Darbinian J Go AS Body mass index and risk for end-stage renal disease Ann Intern Med 2006 144 21 28 16389251 10.7326/0003-4819-144-1-200601030-00006

[B37] Iseki K Ikemiya Y Kinjo K Inoue T Iseki C Takishita S Body mass index and the risk of development of end-stage renal disease in a screened cohort Kidney Int 2004 65 1870 1876 10.1111/j.1523-1755.2004.00582.x 15086929

[B38] Berenson GS Voors AW Dalferes ER Jr Webber LS Shuler SE Creatinine clearance, electrolytes, and plasma renin activity related to the blood pressure of white and black children--the Bogalusa Heart Study J Lab Clin Med 1979 93 535 548 429858

[B39] Voors AW Berenson GS Dalferes ER Webber LS Shuler SE Racial differences in blood pressure control Science 1979 204 1091 1094 10.1126/science.451554 451554

[B40] Fogo A Breyer JA Smith MC Cleveland WH Agodoa LY Kirk KA Glassock R The AASK Study Investigators Renal histopathology in USAfrican-Americans with presumed hypertensive nephrosclerosis Nephrology 1998 4 S54 S58 10.1111/j.1440-1797.1998.tb00473.x

[B41] Ogden CL Flegal KM Carroll MD Johnson CL Prevalence and trends in overweight among US children and adolescents, 1999-2000 JAMA 2002 288 1728 1732 10.1001/jama.288.14.1728 12365956

[B42] Freedman DS Srinivasan SR Valdez RA Williamson DF Berenson GS Secular increases in relative weight and adiposity among children over two decades: the Bogalusa Heart Study Pediatrics 1997 99 420 426 10.1542/peds.99.3.420 9041299

